# *PRELP* Regulates Cell–Cell Adhesion and EMT and Inhibits Retinoblastoma Progression

**DOI:** 10.3390/cancers14194926

**Published:** 2022-10-08

**Authors:** Jack Hopkins, Ken Asada, Alex Leung, Vasiliki Papadaki, Hongorzul Davaapil, Matthew Morrison, Tomoko Orita, Ryohei Sekido, Hirofumi Kosuge, M. Ashwin Reddy, Kazuhiro Kimura, Akihisa Mitani, Kouhei Tsumoto, Ryuji Hamamoto, Mandeep S. Sagoo, Shin-ichi Ohnuma

**Affiliations:** 1 UCL Institute of Ophthalmology, University College London, London EC1V 9EL, UK; 2 Cancer Translational Research Team, RIKEN Center for Advanced Intelligence Project, Tokyo 103-0027, Japan; 3 Division of Medical AI Research and Development, National Cancer Center Research Institute, Tokyo 104-0045, Japan; 4 Department of Ophthalmology, Yamaguchi University Graduate School of Medicine, Yamaguchi 755-8505, Japan; 5 School of Engineering, The University of Tokyo, Tokyo 113-8656, Japan; 6 Retinoblastoma Unit, Barts Health NHS Trust, Royal London Hospital, London E1 1BB, UK; 7 Retinoblastoma Service, Barts Health Trust, Royal London Hospital, London E1 1FR, UK; 8 Department of Respiratory Medicine, Graduate School of Medicine, The University of Tokyo, Tokyo 113-0033, Japan; 9 Institute of Medical Science, The University of Tokyo, Tokyo 108-8639, Japan; 10 NIHR, Biomedical Research Centre for Ophthalmology Moorfields Eye Hospital, London EC1V 2PD, UK

**Keywords:** *PRELP*, retinoblastoma, cell–cell adhesion, dysplasia, EMT/MET

## Abstract

**Simple Summary:**

Mutation of the *RB1* tumor suppressor gene is fundamental in retinoblastoma initiation and progression although its downstream mechanism has not been well elucidated. Here, we found that expression of proline/arginine-rich end leucine-rich repeat protein (*PRELP*) is strongly downregulated in human retinoblastoma and highly expressed in Müller glial cells in normal retina. Deletion of *PRELP* in mice resulted in retinal dysplasia associated with enhanced proliferation. mRNA expression profiling revealed that cancer pathways were strongly activated in *PRELP^−/−^* retina. Additionally, cell–cell adhesion was inhibited while epithelial mesenchymal transition (EMT) and inflammation were activated. On the other hand, application of *PRELP* protein to retinoblastoma cell lines enhances cell–cell and cell–substrate adhesion and inhibits anchorage independent growth by reversing EMT. These observations indicate that *PRELP* downregulation in human retinoblastoma can contribute cancer progression through regulation of cell adhesion and EMT suggested that *PRELP* application might be a novel strategy for retinoblastoma treatment.

**Abstract:**

Retinoblastoma (RB) is the most common intraocular pediatric cancer. Nearly all cases of RB are associated with mutations compromising the function of the *RB1* tumor suppressor gene. We previously demonstrated that *PRELP* is widely downregulated in various cancers and our in vivo and in vitro analysis revealed *PRELP* as a novel tumor suppressor and regulator of EMT. In addition, *PRELP* is located at chromosome 1q31.1, around a region hypothesized to be associated with the initiation of malignancy in RB. Therefore, in this study, we investigated the role of *PRELP* in RB through in vitro analysis and next-generation sequencing. Immunostaining revealed that *PRELP* is expressed in Müller glial cells in the retina. mRNA expression profiling of *PRELP^−/−^* mouse retina and *PRELP*-treated RB cells found that *PRELP* contributes to RB progression via regulation of the cancer microenvironment, in which loss of *PRELP* reduces cell–cell adhesion and facilitates EMT. Our observations suggest that *PRELP* may have potential as a new strategy for RB treatment.

## 1. Introduction

RB is the most common intraocular pediatric cancer with an estimated frequency of 1 in 15,000 to 20,000 live births. RB is aggressive and if left untreated is invariably fatal. Highly specialized clinical interventions including chemotherapy, cryotherapy localized radiotherapy, and enucleation (eye removal) are required to treat RB [1]. These therapies are effective at prohibiting death but often come at the cost of loss of vision in one or both eyes. Moreover, these therapies exert deleterious physical, cognitive, and psychological damage that persists throughout life. Therefore, there is an urgent need to develop more effective and less invasive treatments.

Nearly all cases of RB are associated with mutations compromising the function of the *RB1* tumor suppressor gene. *RB1* was one of the first tumor suppressor genes identified following its cloning in 1986–1987 [2]. *MYCN* hyper amplification without *RB1* mutation has also been reported in a small subset of RB cases [3,4]. Previously, our whole genome sequencing identified at least one *RB1* allele in all 19 RB samples, with several samples also harboring *MYCN* amplification. However, we could not identify any mutation other than *RB1* and *MYCN* [5]. Interestingly, it was thought that loss of *RB1* function results in the initial development of a benign retinoma that later develops into aggressive RB [6]. Furthermore, familial RB patients with *RB1* mutation do not always initiate cancer in both eyes. These observations of incomplete penetrance and phenotypic varaibility of the *RB1* gene indicate that other factors are also involved in RB progression. Indeed, non-mutational epigenetic reprogramming and microenvironmental cues have been shown to drive tumorigenesis in many cancers [7].

*PRELP* is a class II member of the small leucine rich proteoglycan (SLRP) family. SLRPs are highly expressed in ocular tissues and associated with many ocular diseases and cancer [8,9]. These proteins generally reside in the extracellular matrix (ECM) after being secreted, where they were initially recognized to organize and maintain the structural integrity of the ECM via collagen fibrillogenesis. Indeed, *PRELP* is a molecular anchor between constituents of the ECM and the basement membrane [10]. More recent studies have suggested that SLRPs regulate signaling pathways such as TGF-β and Wnt through direct interaction with either ligands or receptors [8]. This can have a profound effect on cancer-specific mechanisms, Recently, we reported that *PRELP* expression was profoundly suppressed in the majority of epithelial cancers [11]. We found that *PRELP^−/−^* mice spontaneously generated benign urothelial papillary cancer, accompanied by a reduction in tight junctions between urothelial epithelial cells that is associated with EGF and TGF-β pathway activation. These changes are quintessential to the EMT phenotype. As *PRELP* is located on chromosome 1q31.1, a region hypothesized to be associated with the initiation of malignancy in RB [6], we investigated the potential role of *PRELP* on the progression of RB through EMT and cell adhesion in this study.

## 2. Materials and Methods

### 2.1. Materials

Antibodies used are listed in Supplemental Table S1. Y79 cells and WERI-RB1 cells were obtained from ATCC and cultured in RPMI-1640 with 10% FBS and 5% L-glutathione. Purified recombinant PRELP were previously reported [12]. Anti-PRELP antibodies were a gift from the Tsumoto laboratory. Conditioned media of *PRELP* was obtained from tetracycline-inducible *PRELP* expressed in HEK293T cells.

### 2.2. Human RB Data

Human RB expression profiling data were obtained in our previous paper [5].

### 2.3. Mouse Experiments

Construction and analysis of the *PRELP^−/−^* mice is described previously [11]. All experiments were covered by Home Office Animal Project Licence and performed in accordance with University College London guidelines for animal handling.

### 2.4. Cell Culture Conditions

Suspension culture: Cells of Y79 or WERI-RB1 were cultured in complete medium in non-treated tissue culture plates (Thermo Fisher Scientific, Waltham, MA, USA, #260860). Where indicated, 1 μg/mL laminin-coated plates were used. Attached culture: Cells were cultured using Nunclon Delta-treated tissue culture plates (Thermo Fisher Scientific, Waltham, MA, USA, #161093).

### 2.5. Viability Assays

First, 0.5 × 10^5^ cells were seeded under either suspension or attached conditions in 96-well plates and incubated at 37 °C for 24 h. Cells were then resuspended in a 1:1 ratio with 0.4% trypan blue. Counting of live and dead cells was performed using a Countess II Automated Cell Counter (Thermo Fisher Scientific, Waltham, MA, USA).

### 2.6. Proliferation and Adhesion Assays

For proliferation assays, 2.0 × 10^4^ cells were cultured under either suspension or attached conditions in 96-well plates and incubated at 37 °C for the indicated time. Then, the CCK-8 assay (ab228554, Abcam, Cambridge, UK) was performed according to the manufacturer’s instructions. A measure of 10 μL of WST-8 reagent was added per 100 μL cell media and incubated for 2 h at 37 °C. Absorbance was then measured at 460 nm. For adhesion assays, 5.0 × 10^4^ cells were seeded in non-treated or 1.0 μg/mL laminin (Sigma, Burlington, MA, USA, L2020) coated plates and incubated for 24 h. Cells were washed twice in PBS and the CCK-8 assay was performed as above.

### 2.7. Anchorage-Independent Growth Assays

First, 0.6% 2-hydroxyethyl agarose was added as a base to 6-well plates and allowed to solidify. In addition, 2.0 × 10^4^ cells were dissociated and suspended in 0.3% 2-hydroxyethyl agarose. Cells were incubated for up to 14 d to form colonies which were imaged at regular intervals using a phase-contrast microscopy. At the end of the experiment, 0.05% crystal violet was used to stain colonies.

### 2.8. Immunostaining

*PRELP^−/−^* retina: Perfusion through direct cardiac approach was used. To analyze dysplasia and other phenotypes, we isolated eye from *PRELP^−/−^* mice and wild type mice at ages of 18–25 weeks as indicated. The isolated eyes were fixed with 4% PFA at room temperature for 1 h and embedded in OCT compound (Tissue Tek, Sakura Finetek, Alphen aan den Rijn, The Netherlands). Then, 10 μm sections were used for X-gal staining and immunohistochemistry as reported [11].

Cultured cells: Cells were fixed for 10 min in methanol for ZO-1 antibody, or in 4% PFA against E-cadherin, N-cadherin, paxillin, and β–catenin and incubated with the primary antibody. Then, cells were stained with the AlexaFluor 488 secondary antibody and visualized by Zeiss LSM 710 confocal microscope.

### 2.9. mRNA Expression Profiling

*PRELP^−/−^* mouse retina: Female wild-type and *PRELP^−/−^* mice aged approximately 13 weeks (*n* = 3) were terminated. RNA was isolated from retinas using RNeasy kits (Qiagen, Manchester, UK). Isolated RNA was assessed using a Nanodrop spectrophotometer (NanoDrop, Wilmington, DE, USA) and an Agilent 4200 Tape Station (Agilent, Santa Clara, CA, USA). mRNA expression profiling of *PRELP^−/−^* mice retina was performed as previously described [11].

Whole cell RNA expression profiling of Y79 cells: 2.0 × 10^6^ Y79 cells were seeded into 6-well plates under suspension conditions. After 24 h culture with 50 μg/mL *PRELP*, RNA was extracted using RNeasy kits and processed as described for the *PRELP^−/−^* mRNA expression profiling.

Single cell expression profiling of WERI-RB1 cells: 0.1 × 10^6^ cells were seeded under suspension conditions in 96-well plates and treated with 50 μg/mL *PRELP* for 16 h. Cells were then dissociated. A total of 12,000 cells from each sample were processed through the 10× Genomics Chromium platform pipeline according to the manufacturer’s instruction. Gel beads in Emulsion were barcoded by oligonucleotides. After RNA quantification with a Qubit 2.0 Fluorometer, sequencing was performed by Illumina NextSeq 2000. Single cell RNA-sequencing was done in duplicate for both control and *PRELP*-treated cells and single cell data were combined to produce a pooled mRNA expression profile to obtain the robust result [13,14].

DESeq2 was used to identify differentially expressed genes which was uploaded to Qiagen Ingenuity Pathway Analysis (IPA) for ontological analysis.

### 2.10. Quantification and Statistical Analysis

Error bars in all graphs indicate standard error of mean. Student *t*-test was used to perform statistical analysis unless stated otherwise. Student *t*-test vales are * = *p*-value < 0.05, ** = *p*-value < 0.005, and *** = *p*-value < 0.001. For in vitro and in vivo experiments, *n* > 3. At least three fields of view were analyzed for each immunofluorescence assay and five fields of view for anchorage-independent growth assay. Statistical analysis was performed using SPSS 27 (version 27.0.1; IBM, New York, NY, USA).

## 3. Results

### 3.1. PRELP Expression Is Strongly Suppressed in RB

Recently, we have performed whole genome and mRNA expression profiling of human RB [5] and investigated the expression of *PRELP* and other members of the SLRP family in human RB (Table 1).

Almost all members of the SLRP family are suppressed in RB compared with the normal retinal tissue. In particular, *PRELP* expression was reduced 163-fold (*p*-value of 1.46 × 10^−22^). We also confirmed low expression of *PRELP* in RB cell lines of Y79 and WERI-RB1 as observed in other cancer cell lines [11]. Our whole genome sequencing analysis confirmed that gene mutations or alternations are largely limited to *RB1* and *NMYC* and SLRP family genes are not mutated [5], indicating that *PRELP* suppression is a consequence of *RB1* mutation in RB.

### 3.2. PRELP Is Highly Expressed by Glial Cells in the Retina

**Figure 1:**
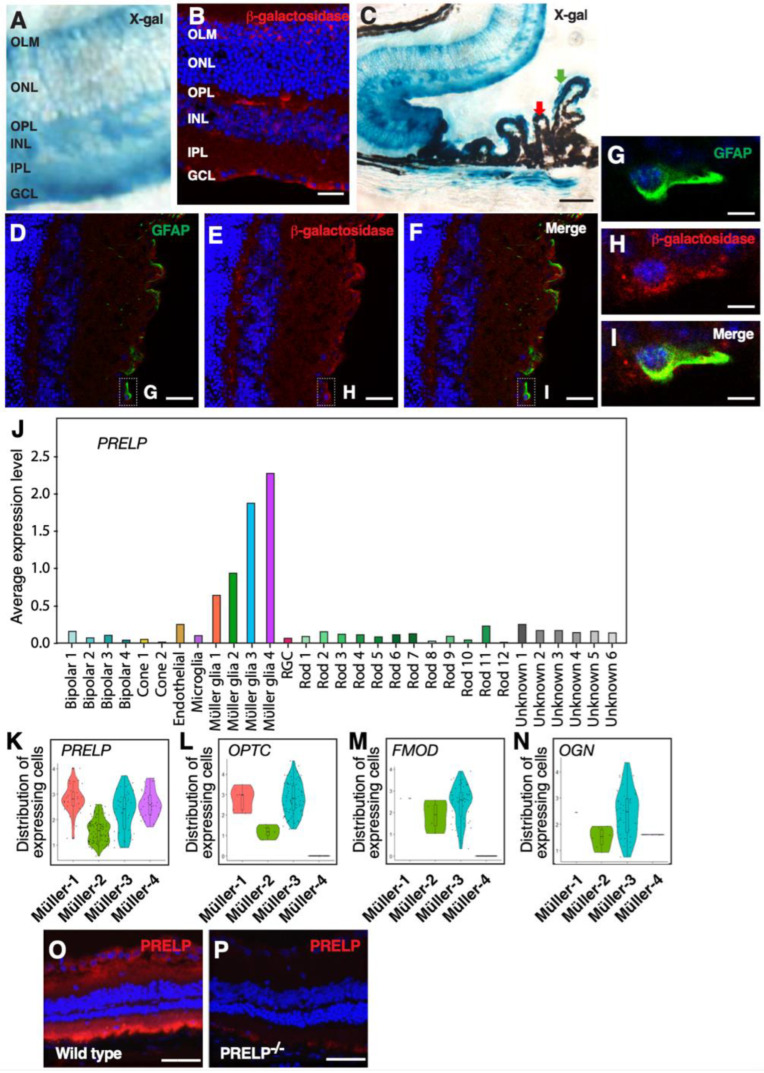
*PRELP* is expressed in Müller glial cells. The blue color indicates DAPI staining: (**A**–**C**) β-galactosidase staining of *PRELP*^−/+^ mouse retina at central area (**A**,**B**) and peripheral retina (**C**). (**A**,**C**) X-gal staining; (**B**) β-galactosidase antibody staining. Scale bar: 50 μm. The staining was seen in around GCL, OPL, basal side of ONL, and OLM in the retina. A stripe staining pattern similar to Müller glial cells was observed. (GCL: Ganglion cell layer, IPL: Inner plexiform layer, INL: Inner nuclear layer, OPL: Outer plexiform layer, ONL: Outer nuclear layer, OLM: Outer limiting membrane, PL: Photoreceptor layer. (**C**) *PRELP* expressed in non-pigmented side of the ciliary body. (**D**–**I**) Double staining of β-galactosidase antibody and anti-GFAP antibody on *PRELP*^−/+^ mouse retina. (**D**) Anti-GFAP staining; (**E**) β-galactosidase antibody staining; (**F**) overlapped image; (**G**–**I**) enlarged image indicated in Figure (**D**–**F**). Scale bar: 10 μm. Clear co-localization was seen on the Müller glial cells on the apical side of the retina; (**J**) Single cell analysis of *PRELP* expression in mouse retina; (**K**) *PRELP* expression in Müller glial cell subtypes; (**L**) opticin expression in Müller glial cell subtypes; (**M**) fibromodulin expression in Müller glial cell subtypes; (**N**) osteoglycin expression in Müller glial cell subtypes; (**O**,**P**) anti-*PRELP* antibody staining of mouse retina (**O**) wild type and (**P**) *PRELP*^−/−^. Anti-*PRELP* antibody staining was seen in almost entire retina. Ganglion cell layer showed intense staining. Scale bar: 50 μm.

After confirmation of *PRELP* suppression in RB, we examined expression patterns of *PRELP* in healthy tissues using *PRELP^−/+^* mice [11]. We first examined endogenous expression of *PRELP* by X-gal staining using a *Lac* reporter gene cloned into *PRELP^−/+^* mice. Expression patterns between the *LacZ* reporter gene in *PRELP^−/+^* mouse and the *PRELP* transcript using in situ hybridization in their wild type equivalents was almost identical [11]. In adult retina, X-gal staining was mainly observed in the retinal ganglion cell layer, the outer plexiform layer, and the basal side of the outer nuclear layer around inner limiting and outer limiting membranes as stripe pattern from apical to basal retina, mimicking Müller glial cell processes (Figure 1A).

The X-gal staining pattern was further confirmed by β-galactosidase antibody staining (Figure 1B). In the peripheral retina, *PRELP* is also expressed at non-pigmented layer of the ciliary body (Figure 1C). To confirm cell types that express *PRELP*, double staining of anti-β-galactosidase monoclonal antibody with Müller glial cell-specific marker GFAP was performed (Figure 1D–I). To determine the Müller glial cell subtypes expressing SLRP family members in the retina, we performed single cell mRNA expression profiling analysis of mouse retina (Figure 1J). Clustering of cell markers identified four subtypes of Müller glial cells. All Müller glial cell clusters expressed *PRELP* (Figure 1K), while other SLRP members were expressed in specific clusters (Figure 1L–N). This is consistent with the literature [15]. *PRELP* localization was determined using monoclonal *PRELP* antibodies [12]. We found that *PRELP* antibody stained the whole retina with the strongest expression in the RGC layer and outer side of photoreceptor cells, which is consistent with our mRNA expression (Figure 1O,P).

**Figure 2:**
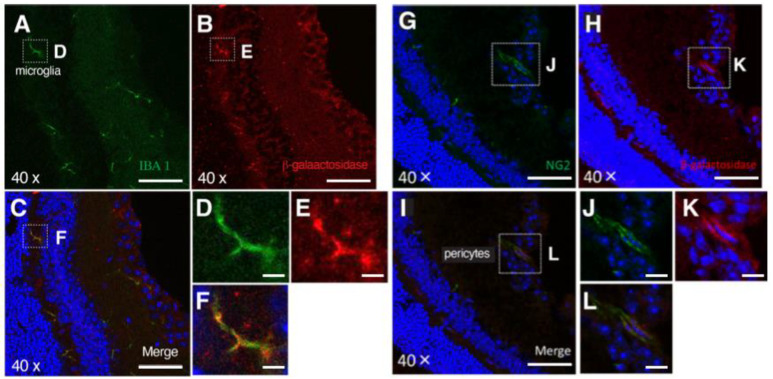
*PRELP* expression in mouse. The blue color indicates DAPI staining: (**A**–**F**) Double staining of (**A**) anti IBA-1 antibody and (**B**) β-galactosidase antibody on *PRELP*^+/LacZ^ mouse; (**C**) merged image. Scale bar: 50 μm. (**D**–**F**) Magnified images. Scale bar: 10 μm. IBA-1 clearly marked microglia in the OPL. Scale bar: 50 μm. (**G**–**L**) Double staining of (**G**) anti-NG-2 antibody and (**H**) β-galactosidase antibody on *PRELP*^+/−^ mouse retina; (**I**) merged image. Scale bar: 50 μm. (**J**–**L**) Magnified images. Scale bar: 10 μm. NG-2 staining marked pericytes tube formation.

Other cell types were also examined, using markers for micoglia (IBA-1), pericytes (NG-2), and vascular endothelial cells (isolectin or green-tomato lectin). We found that in addition to Müller glial cells, a small population of microglia (Figure 2A–F) and pericytes (Figure 2G–L) expressed *PRELP* while no expression was detected in endothelial cells.

### 3.3. PRELP^−/−^ Mice Exhibit Dysplasia in the Retinal Cell Layers

**Figure 3:**
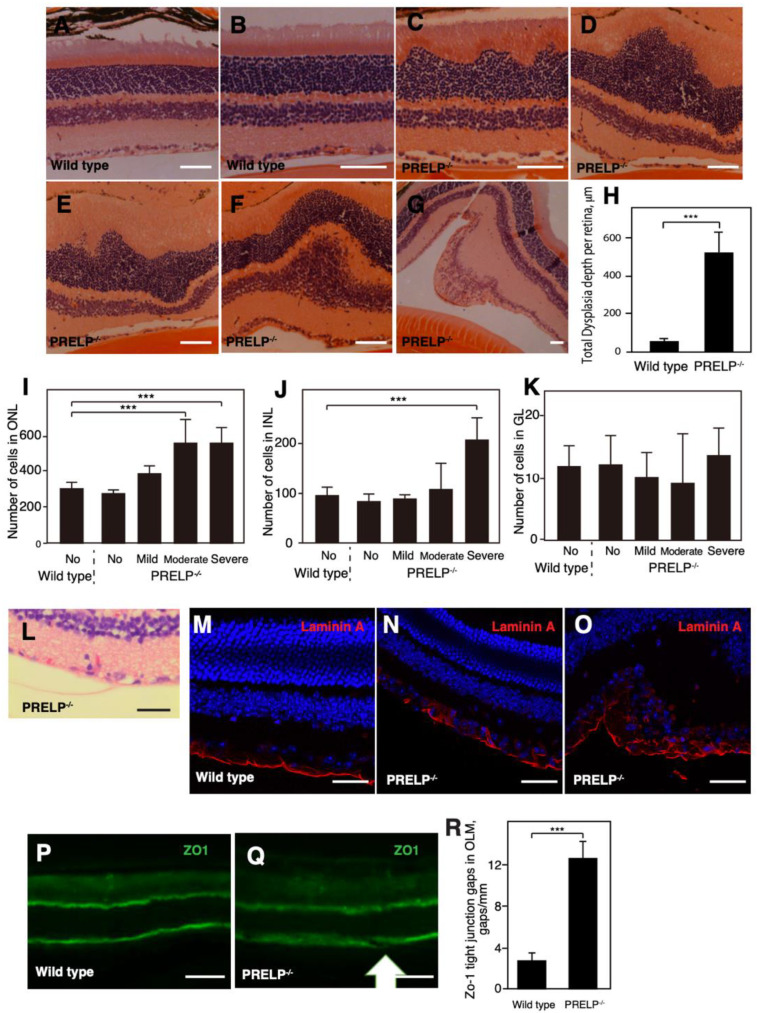
*PRELP* knockout resulted in dysplasia of the retina. The blue color indicates DAPI staining: (**A**–**G**) Hematoxylin and eosin (H&E) staining of retinal section in (**A**,**B**) wild type and (**C**–**G**) *PRELP*^−/−^. Scale bar: 100 μm. In *PRELP*^−/−^ retina, dysplasia was observed in various retinal layers ((**C**–**E**) ONL, (**F**) INL, (**G**) GL). (**H**) Average total dysplasia depth per retina. Retinal dysplasia in wild type and *PRELP^−/−^* retina was quantified. (**I**–**K**) The cell numbers in dysplasia areas and non-dysplasia areas in (**I**) ONL, (**J**) INL, and (**K**) GL were compared; (**L**) detachment of inner limiting membrane/ganglion cell layer in *PRELP*^−/−^ retina. Scale bar: 50 μm. This is similar to idiopathic epiretinal membrane, also observed in (**C**–**E**); (**M**–**O**) Laminin A staining of (**M**) wild type and (**N**,**O**) *PRELP*^−/−^ retina; (**P**–**R**) ZO-1 staining of (**P**) wild type and (**Q**) *PRELP*^−/−^ retina. Scale bar: 100 μm. Gap in outer limiting membrane is indicated as arrow. (**R**) The number of gaps in outer limiting membrane per mm is counted. N ≥ 3. At least three fields of view were analyzed in each experiment. Student’s *t*-test was performed: *** = *p* < 0.001.

We examined the effects of *PRELP* inactivation in the developing retina by comparing retinal morphology in wild type and *PRELP*^−/−^ mice. Wild type mice presented discrete borders between each retinal layers throughout the entire retina (Figure 3A,B). However, architecture in the *PRELP*^−/−^ mouse retina was disrupted (Figure 3C–G). All *PRELP*^−/−^ mice had localized regions of severe dysplasia. This occurred in the outer nuclear layer (Figure 3C–E), inner nuclear layer (Figure 3F), and ganglion cell layer (Figure 3G). Although dysplasia was observed in *PRELP*^−/−^ retina (Figure 3H), most prominently in the outer nuclear layer. We then quantified cell number in these cell layers (Figure 3I–K). No significant differences in cell number at non-dysplastic sites were found. On the other hand, cell number was significantly increased in dysplastic areas, associated with the widening of plexiform layers in some cases. These observations suggest that decreasing cell–cell integrity may enhance proliferative potential, inducing dysplasia.

Abnormalities were also seen in the structure around both inner limiting membrane and outer limiting membrane in *PRELP*^−/−^ mice. In the wild type retina, the structure of the inner limiting membrane was uniform. In the *PRELP*^−/−^ retina, this structure was thinner, more cystic, either hypoplastic or hyperplastic, and often detached (Figure 3C,D). Detachment of the idiopathic epiretinal membrane, which is located at the apical end feet of Müller glial cells and caused by Müller glial cell–mesenchymal transition, was observed in many cases (Figure 3C–E,L) [16]. We stained mouse retina with Laminin A to elucidate ECM structure. Laminin A staining, corresponding to basal membrane, was strongly disrupted in *PRELP*^−/−^ retina (Figure 3M–O). We also stained the tight junction marker, ZO-1, on the outer limiting membrane. In the *PRELP*^−/−^ retina, ZO-1 staining was relatively diffused and disrupted, suggesting that tight junctions in the outer limiting membrane in *PRELP*^−/−^ retina are impaired (Figure 3P–R). These observations indicate that cell–cell adhesion and cellular microenvironment are damaged in *PRELP*^−/−^ retina. Diagnostic hallmarks associated with human RB, such as Flexner–Winersteiner rosetta were not found in *PRELP*^−/−^ mice. This suggests that there is no malignant development of RB in *PRELP^−/−^* mouse retina as observed in human RB.

### 3.4. mRNA Expression Profiling Analysis of PRELP^−/−^ Mouse Retina Revealed That PRELP Is Involved in Cancer, Adhesion, and Inflammation

**Figure 4:**
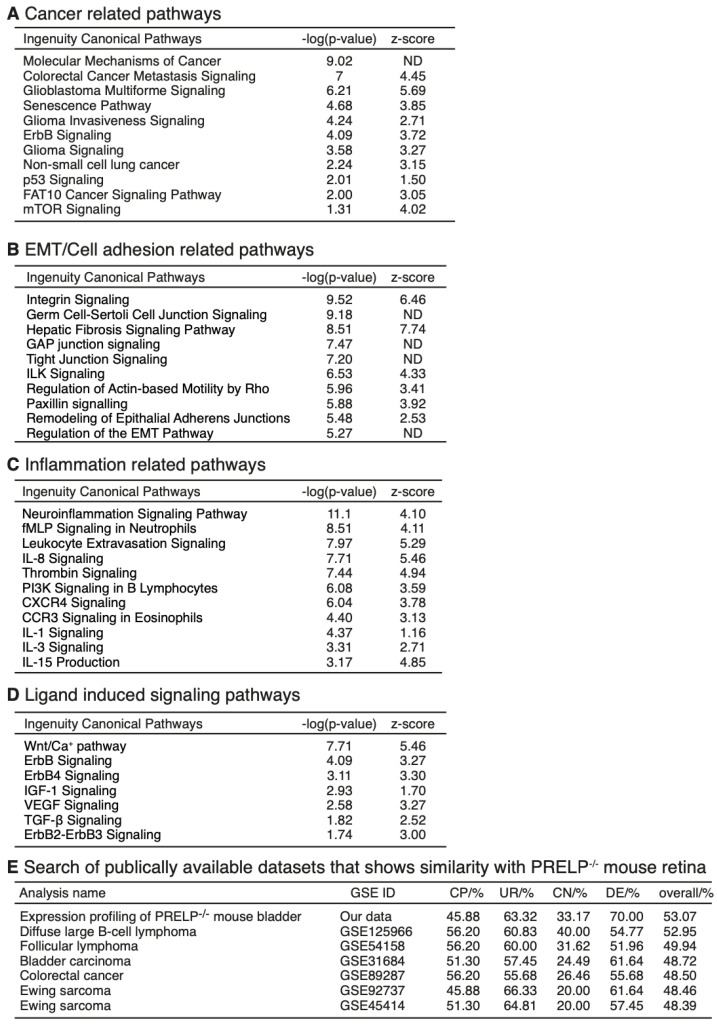
Expression profiling analysis of *PRELP*^−/−^ mouse retina: (**A**) Highly affected cancer−related biological events/pathways in ontological analysis of *PRELP*^−/−^ retina expression profiling data; (**B**) highly affected EMT/cell adhesion−related biological events/pathways; (**C**) highly affected inflammation−related biological events/pathways; (**D**) highly affected ligand−induced signaling pathways; (**E**) search of publicly available datasets that shows similarity with *PRELP*^−/−^ mouse retina. Similar datasets were researched using Analysis Match function of IPA software.

**Figure 5:**
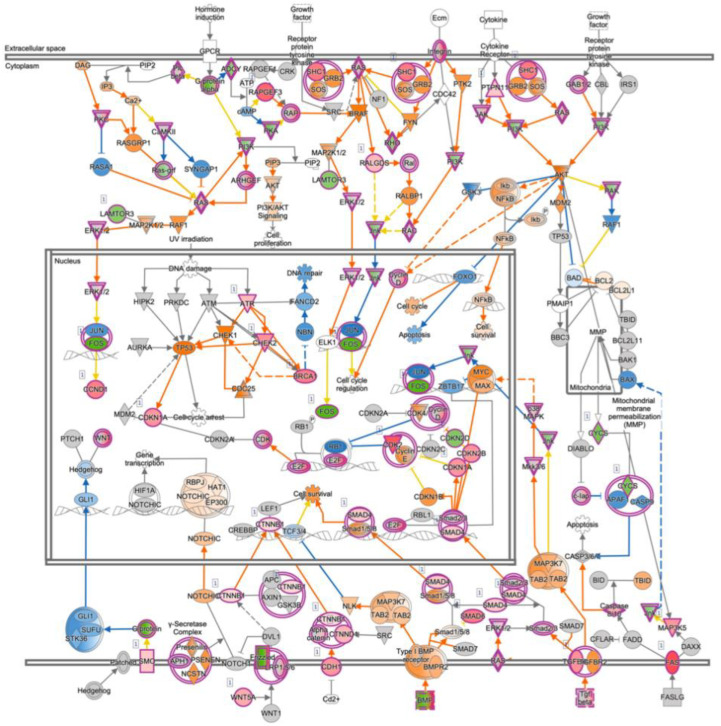
Molecular mechanism of cancer: Schematic drawing of “Molecular Mechanism of Cancer” obtained by ingenuity canonical pathway analysis of *PRELP*^−/−^ mouse retina. Loss of PRELP activated the majority of cancer pathways in the mouse retina. Red = upregulated; green = downregulated; orange = predicted upregulation; blue = predicted downregulation.

To determine the comprehensive effects of the inactivation of *PRELP*, we performed mRNA expression profiling using retinal samples of wild type and *PRELP^−/−^* mice (*n* = 3, female, age 8–12 weeks). Ontological analysis using IPA identified 2051 differentially expressed genes (DEGs) and 276 pathways significantly affected by *PRELP* suppression (*p*-value < 0.01) (Supplemental Data S1). Among these, 42 pathways (19.6%) were directly related to cancer (Figure 4A), including “Molecular mechanisms of cancer” we discuss later (Figure 5), “Colorectal cancer metastasis signaling”, and “Glioblastoma multiform signaling”. In addition to *RB1*, major cancer related genes such as *Ras, NFKB, EGFR, AKT1, JUN/FOS, MYC, APC/TCF3, TP53, MET, PTEN*, and *SRC* were affected (Figure 4B). A further 32 pathways (15.0%, Figure 4B) and 39 pathways (18.2%, Figure 4C) were involved in EMT/cell adhesion and inflammatory processes, respectively. Adhesion pathways potentially contributing to EMT include “Sertoli cell–sertoli cell junction signaling”, “Epithelial adherens junction signaling”, and “Tight junction signaling”, while those related to inflammation include “Neuroinflammation signaling pathway”, “Role of macrophages, fibroblasts and endothelial cells in rheumatoid arthritis”, and “IL-8 signaling”.

Our recent studies showed that *PRELP* directly binds multiple extracellular proteins such as TGF-β, EGF receptor, IGFI receptor, and p75NTR components [11,12]. These pathways are known to regulate cell cycle, EMT, cell adhesion, and inflammation. Our expression profiling of *PRELP*^−/−^ retina shows that these pathways are significantly affected (Figure 4D), supporting that *PRELP*-mediated regulation of these pathways are involved in the mouse retina as observed in bladder. To estimate the contribution of *PRELP* downregulation in human cancer, we biostatistically compared expression profiling data of *PRELP*^−/−^ mouse retina with publicly available data using the Analysis Match function of IPA software (QIAGEN). Figure 4E shows that *PRELP*^−/−^ mouse retina shows very high similarity with publicly available deposited cancer data in all four analysis methods of Canonical Pathways (CP), Upstream Regulations (UR), Causal Networks (CN), and Diseases and Function (DE), indicating that *PRELP* suppression in almost all cancers has significant contribution to the cancer initiation or progression.

### 3.5. PRELP Application Reduces RB Cancer Cell Viability in Association with Enhanced Cell Adhesion, Inhibition of Anchorage Independent Growth, and Facilitation of MET

Our observations suggest that suppression of *PRELP* potentiates RB progression through EMT mechanisms, especially those associated with cell adhesion, as observed in bladder cancer. Thus, we hypothesized that application of *PRELP* protein to RB cells is likely to inhibit RB progression by reversing EMT via mesenchymal epithelial transition (MET). To elucidate the roles of *PRELP* in RB progression, we examined the effect of purified recombinant *PRELP* protein [12] on Y79 and WERI-RB1 RB cell lines. RB cells are known to grow as adhesive or suspension cultures depending on cell culture substrates. We used two substrates: Nunclon Delta-treated plates (attached culture) and non-treated plates (suspension culture). Approximately 18.1% or 25.5% of either Y79 or WERI-RB1 cells attached to non-treated plates, respectively. Almost all RB cells attach to Delta-treated plates.

**Figure 6:**
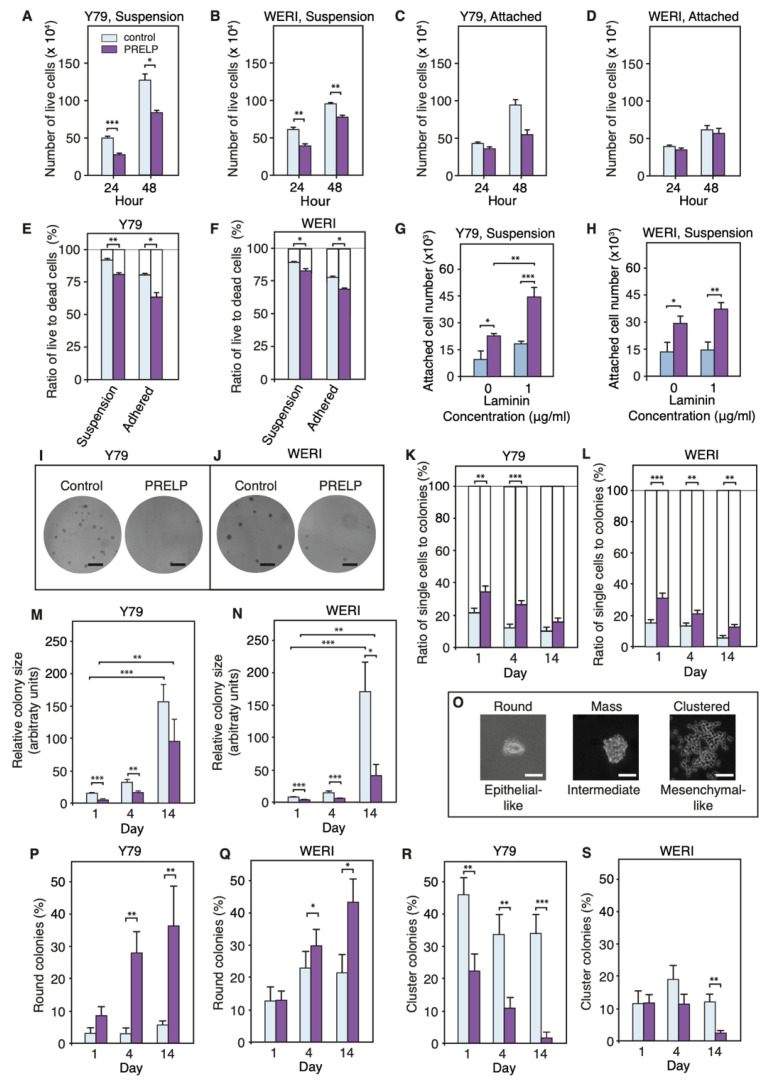
*PRELP* application enhances cell adhesion and inhibits anchorage-independent growth of RB cells: (**A**–**D**) Effect of *PRELP* application on RB cancer cell progression. (**A**) Y79 cells in suspension culture; (**B**) WERI-RB1 cells in suspension culture; (**C**) Y79 cells in attached culture; (**D**) WERI-RB1 cells in attached culture. (**E**,**F**) Effect of *PRELP* on ration of live/dead cells of (**E**) Y79 cells or (**F**) WERI-RB1; (**G**,**H**) Effect of PRELP on cell adhesion. (**G**) Y79 or (**H**) WERI-RB1 were cultured in suspension conditions. The effect of PRELP and laminin A on cell adhesion was examined. (**I**,**J**) Crystal violet staining of (**I**) Y79 cells or (**J**) WERI-RB1 after anchorage-independent conditions. Scale bar: 4 mm. (**K**,**L**) Effect of *PRELP* on ratio of single cells to colonies. (**K**) Y79 cells or (**L**) WERI-RB1; (**M**,**N**) Effect of *PRELP* on colony size in anchorage-independent conditions. (**M**) Y79 cells. (**N**) WERI-RB1. (**O**–**S**) Effect of *PRELP* on formation of three types of colonies (round, mass, and clustered colonies) in anchorage-independent growth conditions. (**O**) Three types colonies. Scale bar: 25 μm. (**P**,**Q**) Percentage of round colonies. (**P**) Y79 cells or (**Q**) WERI-RB1. (**R**,**S**) Percentage of cluster colonies under anchorage-independent conditions. ^®^ Y79 cells or (**S**) WERI-RB1. At least five fields of view were analyzed for anchorage-independent growth studies (*n* = 3). Student’s *t*-test was performed: * = *p*-value < 0.05; ** = *p*-value < 0.01; *** = *p* < 0.001.

We examined the effect of *PRELP* on live RB cell numbers on non-treated plates or Delta-treated plates. Figure 6A,B shows that, on non-treated plates, *PRELP* treatment significantly reduced the number of live cells in both Y79 and WERI-RB1. On the other hand, on Delta-treated plates, effect of *PRELP* on live cell number was weak (Figure 6C,D), suggesting that the effect of *PRELP* depends on cell environment or adhesion status. Next, we examined the effect of *PRELP* on the ratio of live cells to dead cells by trypan blue exclusion. *PRELP* application significantly reduced the ratio of live cells on both non-treated plates and Delta-treated plates (Figure 6E,F), indicating that *PRELP* has negative effect on RB cell survival. Then, we examined the effect of *PRELP* on cell–substrate adhesion. *PRELP* application to Y79 or WERI-RB1 cells strongly enhanced cell adhesion to non-treated plates. A 1 μg/mL laminin treatment enhanced cell adhesion to the substrate and *PRELP* application further enhanced adhesion (Figure 6G,H). The enhancement of adhesion by *PRELP* must be related by its biological function because addition of BSA (50 μg/mL) did not alter the adhesion. The observation that *PRELP* can enhance adhesion of Y79 and WERI-RB1 cells to non-treated or laminin-treated plates suggests that *PRELP* treatment may inhibit cancer cell growth through inhibition of anchorage-independent growth. Therefore, we examined the effect of *PRELP* under anchorage-independent growth conditions. In the absence of *PRELP*, Y79 cells and WERI-RB1 cells developed many large colonies. Addition of *PRELP* strongly inhibited colony formation (Figure 6I,J). We counted the number of colonies and single cells (Figure 6K,L). These observations indicate that *PRELP* inhibits anchorage independent growth of solo RB cells. Furthermore, we quantified relative size of colonies (Figure 6M,N), indicating that *PRELP* reduces the colony size. These corroborate our recent study in bladder cancer using EJ28 bladder cell lines [11]. Our results suggest that *PRELP* has tumor suppressor-like properties through regulation of EMT and cell adhesion in many types of cancer.

**Figure 7:**
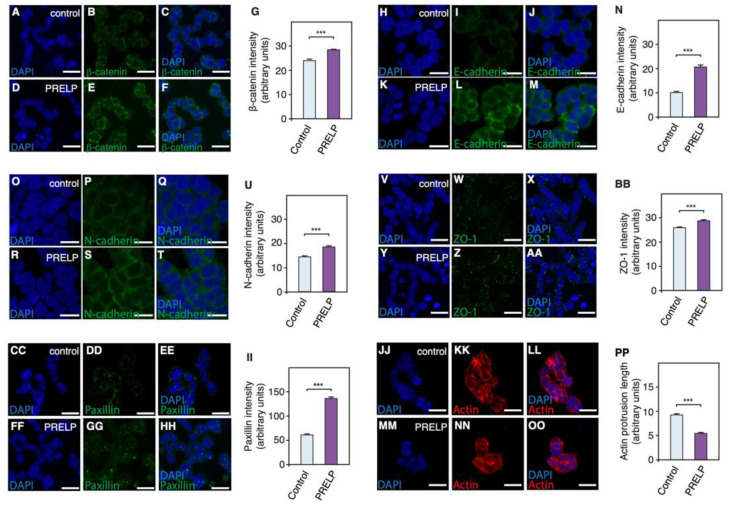
Application of PRELP resulted in conversion from mesenchymal cells to epithelial cells and change of adhesive properties: Y76 cells were cultured in suspension conditions and then the effect of PRELP application on various cell adhesion and EMT markers. (**A**–**G**) Effect on β-catenin staining. (**H**–**N**) Effect on E-cadherin staining. (**O**–**U**) Effect on N-cadherin staining. (**V**–**BB**) Effect on ZO-1 staining. (**CC**–**II**) Effect on Paxillin staining. (**JJ**–**PP**) Effect on Actin staining. N = 3. At least five fields of view were analyzed for each experiment (*n* = 3). Student’s *t*-test was performed: *** = *p* < 0.001. Scale bar: 10 µm.

Anchorage-independent growth has been previously shown to produce distinct colony types, which correspond to EMT states and contribute to metastatic potential. As observed in breast cancer [17], there were three distinct colony types of RB cells in our anchorage-independent growth assays (Figure 6O). Round colonies were highly organized, with a discrete boundary. Mass colonies displayed a lack of organization while clustered colonies were disseminated. These phenotypes have been previously demonstrated to be spaced along the EMT spectrum, with round colonies presenting epithelial-like qualities and clustered colonies demonstrating mesenchymal-like qualities. Application of *PRELP* to RB cell lines increased the proportion of round colonies (Figure 6P,Q) whilst decreasing the number of clustered colonies (Figure 6R,S), indicating that *PRELP* application resulted in MET. Next, we examined the effect of *PRELP* application on expression of adherens junctions (β-catenin, E-cadherin, N-cadherin) and tight-junction (ZO-1). *PRELP* application enhanced β-catenin, E-cadherin, and N-cadherin staining at the plasma membrane (Figure 7A–U). ZO-1 staining was also enhanced by *PRELP* application (Figure 7V–BB), indicating that *PRELP* indeed activate cell–cell adhesions. We also examined the effect of PRELP on paxillin and actin. *PRELP* treatment resulted in diffused paxillin staining (Figure 7CC–II). In control, long and clear actin fibers were observed, which were reduced with PRELP treatment (Figure 7JJ–PP) *PRELP* application clearly enhanced epithelial type staining and weakens mesenchymal type staining.

### 3.6. Expression Profiling after Application of PRELP Revealed That PRELP Suppresses Various Tumor Related Pathways and Enhances Cell-Cell Adhesion

**Figure 8:**
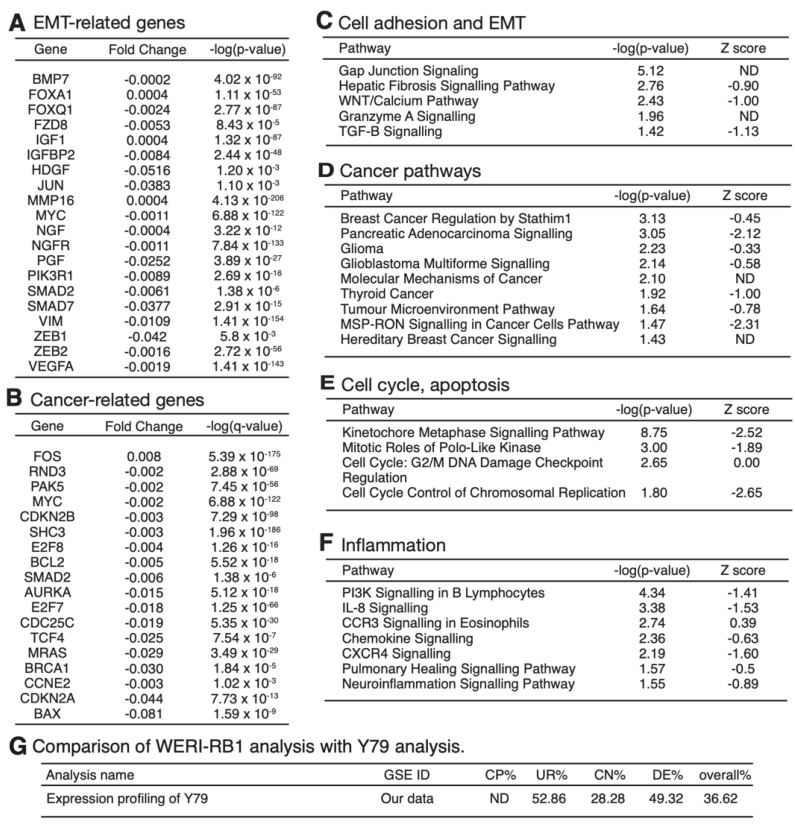
PRELP protein application to WERI−RB1 RB cells and the expression profiling analysis: (**A**,**B**) Sorted by fold change. (**A**) Strongly affected EMT−related genes by PRELP application; (**B**) strongly affected cancer−related genes; (**C**–**F**) sorted by *p*−value. (**C**) Strongly affected cell adhesion/EMT-related pathways; (**D**) strongly affected cancer−related pathways; (**E**) strongly affected cell cycle/apoptosis−related pathways; (**F**) strongly affected inflammation−related pathways; (**G**) comparison of WERI-RB1 analysis with Y79 analysis by Analysis Match function of IPA software.

To elucidate comprehensive action mechanism of *PRELP* on RB, we applied *PRELP* protein to Y79 or WERI-RB1 cells and performed mRNA expression profiling. In the case of WERI-RB1, the cells were cultured as suspension condition and then the effect of *PRELP* (50 μg/mL) was examined by single-cell based bulk mRNA expression profiling as indicated in Experimental Procedures (Supplemental Data S2). Firstly, we examined expression of EMT-related genes and cancer-related genes. Many of EMT-related genes and cancer-related genes were strongly suppressed after *PRELP* application (Figure 8A,B). Then, we performed ontological analysis using IPA software. Cell adhesion pathways are strongly activated, while EMT related pathways are inhibited, supporting our in vitro observations (Figure 8C). Cancer pathways are strongly inhibited by *PRELP* (Figure 8D).

**Figure 9:**
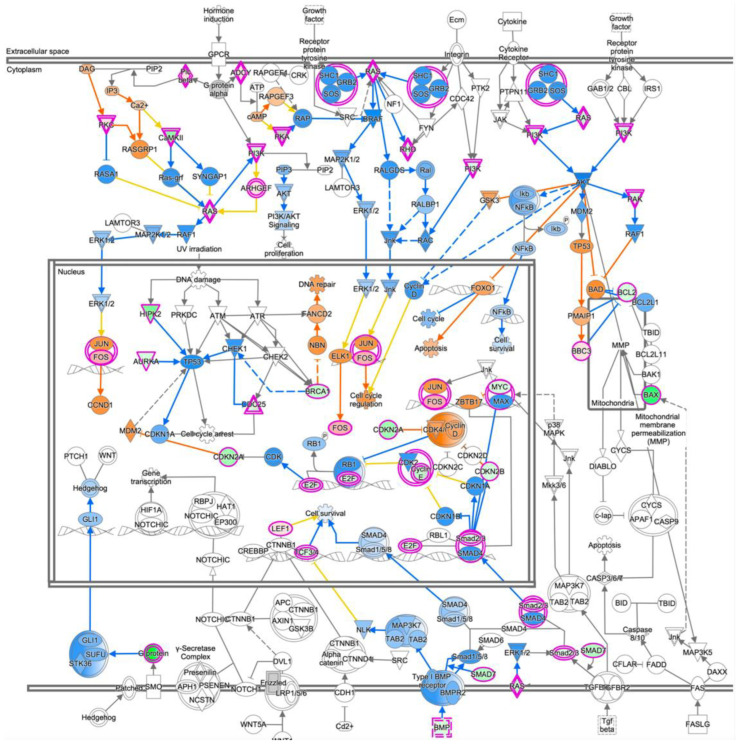
Molecular mechanism of cancer: Schematic drawing of “Molecular Mechanism of Cancer” obtained by ingenuity canonical pathway analysis of PRELP application to WERI-RB1 cells. The majority of cancer-associated pathways were downregulated in PRELP-treated WERI-RB1 cells. Red = upregulated; green = downregulated; orange = predicted upregulation; blue = predicted downregulation.

Figure 9 shows a drawing of “Molecular Mechanism of Cancer”, indicating that many oncogenic pathways such as RAS, NFκB, and AKT are negatively regulated by *PRELP*. Furthermore, cell cycle is inhibited, while apoptosis is activated. TGF-β pathway and growth factor pathways are inhibited by *PRELP* as observed in biochemical analysis on bladder cancer cells [11]. Figure 9 shows strong contrast to that of *PRELP^−/−^* mouse retina analysis (Figure 5), confirming the role of *PRELP* in cancer progression. Figure 8E shows that PRELP inhibits cell cycle and potentiates cell death. *PRELP* also inhibits inflammation-related pathways (Figure 8F). We also performed standard bulk expression profiling analysis using Y79 cells as indicated in Experimental Procedures (Supplemental Data S3). As expected, PRELP application to Y79 cells affected cell adhesion, EMT-related pathways, cancer pathways, cell cycle, and inflammation as observed in WERI-RB1 cells. We compared similarity between WERI-RB1 cell analysis and Y79 cell analysis by Analysis Match. Figure 8G clearly indicates high similarity of these two analyses.

## 4. Discussion

Enhanced proliferative capacity and remodeling of cell adhesive property are major characteristics of cancers. Here, our analysis of human RB expression profiling and subsequent analysis of *PRELP^−/−^* mouse retina identified that suppression of *PRELP* contributes to weakening cell adhesive property and facilitating EMT in RB, which enhances progression of RB. Furthermore, we demonstrated that *PRELP* application activates cell–cell adhesion and MET and suppresses viability of RB cell lines. These observations indicate the importance of regulation of cell adhesive property by controlling microenvironment in cancer. *PRELP* may have a fundamental role in the microenvironment and seems to function as a novel type of a tumor suppressor against RB.

### 4.1. PRELP Secreted from Müller Glial Cells Has Function to Maintain Rigid Retinal Structure

The retina is an immune privileged tissue and is against tumor development even when exposed to UV radiation in daily life, which is a strong carcinogen. The retina is rigidly packed into distinct layers, which are important for the highly coordinated retinal functions. This rigid layered structure of the retina is maintained by a wide range of mechanisms. In particular, Müller glial cells have important roles through providing structural frameworks in the retina and also providing secreted proteins that contribute to cell–cell adhesion [18,19,20]. Our results indicate that *PRELP* is selectively expressed by Müller glial cells. Furthermore, deletion of *PRELP* in mice clearly leads to retinal dysplasia and down regulation of many adhesion-related biological events. Moreover, we observed that detachment of inner limiting membrane as observed in idiopathic epiretinal membrane in *PRELP^−/−^* mouse retina. Idiopathic epiretinal membrane is located around the apical end feet of Müller glial cells and is caused by TGF-β-Snail pathway-mediated Müller glial cell–mesenchymal transition [16], suggesting that *PRELP* deletion induced EMT in Müller glial cells themselves. Interestingly, idiopathic epiretinal membrane is associated with myopia [16]. Since *PRELP* is highly expressed in sclera [21] and regulated EMT [11], *PRELP* might be aassociated with myopia [22]. These observations indicate that *PRELP* is one of the molecules that contribute to maintenance of retinal structure.

How does the dysplasia of retina with localized activation of proliferation occur? There are two potential explanations; (1) the damage of cell–cell adhesion firstly initiated dysplasia and then localized proliferation was activated around the dysplasia or (2) abnormal proliferation resulted in dysplasia. We observed that the increased number of cells is only observed in dysplasia regions, suggesting that defect of cell adhesion damage is required for activation of proliferation. Our analysis of molecular mechanism indicates that PRELP can regulate at least three signaling pathways of TGF-β, EGF, and the canonical Wnt pathways [11]. Our expression profiling analysis indicate that all these signaling seems to be affected in *PRELP^−/−^* retina and human RB. These pathways are major pathways in the regulation of EMT [23,24]. Indeed, EMT is strongly affected in *PRELP^−/−^* retina. In addition, these pathways are well known as major oncogenic pathways [25,26,27,28]. These observations indicate that the defect of PRELP mediated activation of cell–cell adhesion and MET through regulation of TGF-β, EGF, and the canonical Wnt pathways is the major cause of dysplasia in *PRELP^−/−^* retina.

### 4.2. Role of PRELP-Depleted Microenvironment in RB Progression

We have found that *PRELP* expression is strongly downregulated in RB (Table 1) and that downregulation of PRELP revealed important roles in progression of RB as we demonstrated in this study. We previously discovered the downregulation of *PRELP* in many different cancers, including retinoblastoma [11]. We revealed that loss of PRELP is an important prerequisite for bladder cancer onset using *PRELP^−/−^* mouse. In this study, only four SLRPs had a fold change less than −100. Of these, NYX and OGN expression levels were 80.0 and 1584.3, respectively. PRELP and OPTC expression levels were 4102.7 and 5104.3 respectively, indicating that these proteins could be more important in normal retina function. Additionally, the *PRELP* gene is located on chromosome 1q31.1, which is a site frequently perturbed in cancer. The comparison of expression profiling data of *PRELP^−/−^* retina and human cancers has revealed that influenced mechanisms and genes in *PRELP^−/−^* retina are highly conserved with cancer progression mechanisms in other cancers (Figure 4E), indicating that the PRELP downregulation in human RB contributes to the progression through regulation of the pathways regulated by PRELP. In addition, expression profiling analysis of *PRELP^−/−^* mouse retina indicated that PRELP itself has ability to regulate cancer-related pathways, cell adhesion, and EMT without RB1 abnormality. Regulation of cell–cell adhesion in cancer is fundamental. Anchorage independent growth is a hallmark of cancer cells [29,30]. EMT, which contains loss of cell–cell adhesion, is required for cancer initiation and progression [31]. In general, less adhesive cancer is more malignant. RB is a very malignant type of cancer. Reflecting this, cancer cell lines originated from RB are not adhesive, requiring suspension culture. The importance of regulation of cell–cell adhesion in RB progression is clearly demonstrated in this study, in which PRELP has fundamental roles in the regulation of cell–cell adhesion and inhibiting RB cell progression.

In RB, the importance of cell–cell adhesion was previously demonstrated by CDH11 study [32]. CDH11 is a type II classical cadherins family, which regulate cell–cell adhesion. Marchong et al. showed that *CDH11* gene copy number and expression are frequently lost in human RB. Crossing of *Cdh11* null mice with TAg-RB, RB model mice, resulted in faster tumor growth than Tag-RB alone through downregulation of tumor cell death, proposing that CDH11 works as a tumor suppressor through activation of cell–cell interaction. CDH11 is reported to inhibit the canonical Wnt pathway through downregulation of β-catenin [33]. However, our expression profiling data of human RB shows that *CDH11* is not significantly suppressed in RB [5]. Furthermore, in seven RB that we analyzed, we did not identify mutation or amplification of *CDH11* [5]. In addition, our expression profiling analyses of *PRELP^−/−^* mice and PRELP overexpression show that the expression of *CDH11* was not significantly affected, indicating that PRELP and CDH11 are important for cell–cell adhesion through interaction with the canonical Wnt pathways but work under distinct mechanisms in tumor suppression.

Moreover, the molecular function of PRELP as a regulator of major cancer-related pathways of TGF-β, EGF, and Wnt also strongly indicates that PRELP suppression is not just consequence of RB development. *PRELP* is located at 1q31.1, where it is associated with transition from retinoma to RB [6]. Our study strongly suggests that PRELP downregulation may have an important role in RB progression. We found that the upregulation of both N-cadherin and E-cadherin in PRELP-treated cells indicated that the two cadherins are regulated by PRELP. Although the upregulation of N-cadherin is associated with EMT, our in vitro assays demonstrated that PRELP application enhanced cell adhesion and suppressed anchorage-independent growth of RB cells. These findings suggest that PRELP altered oncogenic and tumor suppressive cadherin expression coincidentally, but tumor suppressive roles are dominant.

To link these processes to *RB1* mutation, genes directly under expression control of RB1 or E2Fs (de-repressed by *RB1* mutation) were analyzed. *RB1* loss-of-function directly reduced expression of a cadherin family member, *CELSR1*, linked to adherens junction stability [34]. E2F1 de-repression also induced *ECT2* expression; an oncogene encoding a RhoA-activating GEF linked to NSCLC cell proliferation and invasion [35]. *RB1* mutation may directly impair immune cell recruitment and contribute to reduced inflammation, as several immune cell adhesion molecules were reduced by E2F1 de-repression (*SELE*, *ICAM1*, *TAPBP*). Several additional regulators of chromatin structure and transcription were directly enhanced by RB1 loss-of-function and/or E2F activation, including *RBBP4*, a ubiquitous component of histone deacetylase complexes that was upregulated in an *RB1*-mutation-driven embryonic brain tumor model in zebrafish [36] and is known to enhance mesenchymal marker expression and invasion in human cervical and colon cancer cell lines [37,38].

### 4.3. Difference between PRELP^−/−^ Mouse Retina and Human RB

Our analysis of *PRELP^−/−^* mouse retina showed dysplasia-associated activation of proliferation. However, mice did not develop malignancies, as observed with human RB. This observation indicates that *RB1* mutation and/or *MYCN* amplification can regulate PRELP-independent mechanisms that are important for proper development of malignant RB.

### 4.4. Generation of PRELP-Depleted Microenvironment in RB

Our results indicate that PRELP suppression is important for RB progression. However, there are two possible explanations of how the low expression is established. (1) PRELP expression is indeed suppressed in the cancer cells during RB progression. If so, this suppression must be under transcriptional control because our previously published whole genome sequencing did not identify any mutations or deletion of the *PRELP* gene [5]. Interestingly, RBBP4, a downstream regulator of RB1, is upregulated by RB1 mutation [36] and enhances mesenchymal marker expression in human cervical and colon cancer cell lines [37,38]. (2) If RB is not formed from Müller glial cells, exclusion of Müller glial cells from cancer tissue because of expansion of cancer cells might be the mechanism as reduction of PRELP expression in RB. To identify which mechanism is correct, the cell of origin for RB is important. However, the cell of origin has not been clearly determined [39]. Amacrine neurons and horizontal neurons are reported to initiate RB-like cancer in *RB1/p107* mouse model and *RB1/p130* mouse model, respectively [40,41,42]. *PRELP* is expressed largely in Müller glial cells in mice. In *RB1/p107* and *RB1/p130* models, some cancer cells were positive to Müller glial cell markers. Müller glial cells have proliferative potential properties [43,44]. Cone photoreceptor cells are reported as a candidate of cell of origin in human iPS cell-based model [7]. Recently, Norrie et al. demonstrated that iPS cells originated from RB patients can initiate RB after xenograft into mouse eyes. Single cell analysis of RB development process shows that the RB had mixed cell type properties [45]. This is consistent with expression profiling of human RB. Furthermore, our *PRELP*^−/−^ retina caused dysplasia in both the INL and ONL. Further study is required to determine RB cell of origin. Although cell of origin is important for understanding RB, understanding of the autonomous PRELP suppression mechanisms may not be very important because PRELP is a secreted protein and influences cancer progression via microenvironment.

### 4.5. ECM in Cancer

Secreted PRELP protein distributes largely around the apical side of Müller glial cell end-feet but widely defuses over the retina. ECM proteins have fundamental roles in various aspects in cancer initiation and progression [46,47]. In particular, they create a complex microenvironment for cancer progression [48]. The importance of ECM proteins was clearly observed when overexpression of MMP3 in mice induced mammary carcinoma, despite having cancer-associated gene mutation [49]. Some SLRP family members are known substrates of MMPs [50,51], and Type-I SLRPs, such as Decorin and Biglycan have been reported to affect cancer progression [52]. This paper demonstrates that the Type-II SLRP member, PRELP, is involved in RB progression. ECM proteins, including PRELP are important in regulating ocular tissues This paper and our previous paper [11] propose that PRELP is an important ECM protein in the initiation and progression of various cancers through control of cell–cell. PRELP-mediated improvement of the cancer microenvironment might be a better strategy for treatment of RB.

## 5. Conclusions

In this paper, we demonstrated the functional roles of PRELP, which are the association with the inhibitory effects for RB through regulating cell–cell adhesion, EMT, and inflammatory signaling. Moreover, exogenously supplied PRELP protein to RB cells showed enhancement of cell-cell and cell-substrate adhesion and the inhibition of anchorage-independent growth by reversing EMT. Therefore, PRELP may have potential as an anti-cancer drug to treat RB patient in the future.

## Figures and Tables

**Table 1:** Expression levels of SLRP family in human RB.

SLRP Member	Normal Retina Expression (*n* = 3)	RB Expression (*n* = 3)	Fold Change	*p*-Value
Osteoglycin (OGN)	1584.3	7.8	−203.1	1.1 × 10^−97^
Opticin (OPTC)	5104.3	0.6	−8507.2	3.4 × 10^−41^
Osteomodulin (OMD)	318.7	1.8	−177.0	8.7 × 10^−32^
Proline/arginine-rich end leucine-rich repeat Protein (*PRELP*)	4102.7	25.2	−162.8	1.5 × 10^−22^
Nyctalopin (NYX)	80.0	0.8	−100.0	5.6 × 10^−14^
Podocan−like protein 1 (PODNL1)	190.0	7.4	−25.7	7.0 × 10^−10^
Tsukushi (TSKU)	983.3	219.6	−4.5	1.4 × 10^−7^
Biglycan (BGN)	1361.7	123.0	−11.1	6.1 × 10^−7^
Fibromodulin (FMOD)	620.3	31.8	−19.5	6.5 × 10^−7^
Asporin (ASPN)	38.3	7.4	−5.2	4.6 × 10^−5^
Extracellular matrix protein 2 (ECM2)	203.3	14.6	−13.9	1.4 × 10^−4^
Decorin (DCN)	1785.7	87.0	−20.5	1.8 × 10^−4^
Chondroadherin (CHAD)	12.0	0.8	−15.0	2.5 × 10^−3^
Keratocan (KERA)	12.7	1.0	−12.7	5.7 × 10^−2^
Lumican (LUM)	98.0	20.2	−4.9	1.1 × 10^−1^
Podocan (PODN)	163.0	123.4	−1.3	NA
Epiphycan (EPYC)	0	0	NA	NA

## Data Availability

The data presented in this study are available in the supplementary materials and are available from the corresponding author upon reasonable request.

## Supplementary Materials

The following supporting information can be downloaded at: https://www.mdpi.com/article/10.3390/cancers14194926/s1, Table S1: List of antibodies; Data S1: Signaling pathways and biological functions regulated genes in PRELP^−/−^ retina; Data S2: Signaling pathways and biological functions significantly affected after application of PRELP protein to WERI-RB1 cells; Data S3: Signaling pathways and biological functions significantly affected after application of PRELP protein to Y79 cells.
