# Supplementary material for: PRELP Regulates Cell–Cell Adhesion and EMT and Inhibits Retinoblastoma Progression
Source: Cancers (Basel). 2022 Oct 8;14(19):4926. doi: 10.3390/cancers14194926 (PMC9563430; doi:10.3390/cancers14194926)
Supplement: Supplementary file 1 [file cancers-14-04926-s001.zip › Supplemental Table S1.pdf]

Supplemental Table S1

List of antibodies

Antibody/reagent	Manufacturer	Cat Number	Dilution
β-galactosidase	Millipore	559761	1:100
Anti-NG2	Millipore	Ab5320	1:100
Anti-Iba-1	Abcam	ab178846	1:100
Anti-GFAP	Sigma-Aldrich	G4546	1:100
Anti-SMA	Abcam	ab5694	1:100
Anti-laminin	Abcam	ab11575	1:100
Anti-ZO-1	Invitrogen	402300	1:200
Anti-β-catenin	CST	8480	1:100
Anti-E-cadherin	Abcam	ab1416	1:100
Anti-N-cadherin	CST	13116T	1:100
Anti-Paxillin	Abcam	ab32084	1:100
Rhodamine-Phalloidin	Thermofisher	R415	1:2000
Anti-Rabbit Alexa 488	Invitrogen	A11008	1:1000
Anti-Rabbit Alexa 594	Invitrogen	A11012	1:1000
Anti-Mouse Alexa 488	Invitrogen	A11054	1:1000
Anti-Mouse Alexa 594	Invitrogen	A11005	1:1000
Lycopersicon Esculentum (Tomato) Lectin, DyLight® 594	Vector Labs	FL-1171-1	1:500
Lycopersicon Esculentum (Tomato) Lectin, DyLight® 488	Vector Labs	DL-1174-1	1:500
